# Candidate genes for idiopathic epilepsy in four dog breeds

**DOI:** 10.1186/1471-2156-12-38

**Published:** 2011-04-25

**Authors:** Kari J Ekenstedt, Edward E Patterson, Katie M Minor, James R Mickelson

**Affiliations:** 1 Department of Veterinary Clinical Sciences, College of Veterinary Medicine, University of Minnesota, 1352 Boyd Avenue, Saint Paul, Minnesota, 55108, USA; 2 Department of Veterinary and Biomedical Sciences, College of Veterinary Medicine, University of Minnesota, 295 AS/VM, 1988 Fitch Avenue, St. Paul, Minnesota, 55108, USA

## Abstract

**Background:**

Idiopathic epilepsy (IE) is a naturally occurring and significant seizure disorder affecting all dog breeds. Because dog breeds are genetically isolated populations, it is possible that IE is attributable to common founders and is genetically homogenous within breeds. In humans, a number of mutations, the majority of which are genes encoding ion channels, neurotransmitters, or their regulatory subunits, have been discovered to cause rare, specific types of IE. It was hypothesized that there are simple genetic bases for IE in some purebred dog breeds, specifically in Vizslas, English Springer Spaniels (ESS), Greater Swiss Mountain Dogs (GSMD), and Beagles, and that the gene(s) responsible may, in some cases, be the same as those already discovered in humans.

**Results:**

Candidate genes known to be involved in human epilepsy, along with selected additional genes in the same gene families that are involved in murine epilepsy or are expressed in neural tissue, were examined in populations of affected and unaffected dogs. Microsatellite markers in close proximity to each candidate gene were genotyped and subjected to two-point linkage in Vizslas, and association analysis in ESS, GSMD and Beagles.

**Conclusions:**

Most of these candidate genes were not significantly associated with IE in these four dog breeds, while a few genes remained inconclusive. Other genes not included in this study may still be causing monogenic IE in these breeds or, like many cases of human IE, the disease in dogs may be likewise polygenic.

## Background

The prevalence of epilepsy in humans is reported to be in the range of 4 - 10/1000 in most study settings [1], with idiopathic epilepsy (IE) representing 15 - 20% of these cases [2]. It is now generally accepted that IE in humans is due to an underlying genetic origin [2], although causative mutations have been discovered in only a small subset of IEs, mostly in isolated populations. These identified epilepsy mutations are, for the most part, Mendelian or monogenic IEs [3-6], and are often termed "channelopathies" due to their occurrence in ion channel genes. In a recent review, 16 of 21 susceptibility genes for human epilepsy were ion channels or neurotransmitter receptors [7].

Canine epilepsy is a naturally occurring, spontaneous condition. Canine seizures exhibit a remarkable resemblance to human seizures [8] and the usefulness of naturally occurring canine epilepsy as a translational model to explore potential treatments for human epilepsy was recently proposed by Leppik, et al. [9]. The prevalence of canine epilepsy is estimated to be between 0.5% and 5.7% and it is the most common chronic neurological disorder in dogs [10]. A diagnosis of IE in the canine indicates recurrent seizures for which no cause can be identified and implies a genetic predisposition [11]. While the molecular basis of IE in canines is entirely unknown, a hereditary basis for IE has been suggested in a number of dog breeds including Beagles [12], British Alsatians [13], Keeshonds [14], Labrador retrievers [15], Golden retrievers [16,17], Bernese mountain dogs [18], Belgian Tervurens [19,20], Boxers [21], Shetland sheepdogs [22], Vizslas [23], English Springer Spaniels [24], Irish wolfhounds [25], and Standard Poodles [26]. One inherited progressive seizure disorder that has been identified in dogs is the autosomal recessive Lafora disease in Miniature Wirehaired Dachshunds [27]. This progressive myoclonic epilepsy disorder results from a mutation in the *EPM2B *(also known as *NHLRC1*-NHL repeat containing 1) gene.

Modes of inheritance predicted from examination of pedigrees of dogs with IE show evidence for a gene of major effect or autosomal recessive inheritance, both of which could occur due to a founder effect. Examples include the English Springer Spaniel (partially penetrant autosomal recessive or polygenic inheritance) [24], the Vizsla (autosomal recessive or polygenic inheritance) [23], and the Golden Retriever [16] and the Bernese Mountain Dog [18] (both autosomal recessive but controlled by more than one gene). There is enough variation in mode of inheritance and clinical characteristics in dogs to presume that the underlying genetic basis is unlikely to be identical between breeds.

Linebreeding and inbreeding have become commonplace to meet the very specific dog breed standards of the hundreds of dog breeds in existence today. These practices, while creating more uniform offspring, can also result in deleterious diseases due to the concentration of recessive mutations passed from the founders. Almost half of the nearly four hundred genetic disorders that have been described in dogs are recognized exclusively in one or a few breeds [28], presumably due to a strong founder effect.

The aim of the present study was to take advantage of the likely founder effect occurring in purebred dogs with IE in order to investigate an underlying genetic basis for IE in four dog breeds: the Vizsla, the English Springer Spaniel (ESS), the Greater Swiss Mountain Dog (GSMD) and the Beagle. A candidate gene approach was utilized; genes known to be involved in inherited human epilepsy, along with two genes involved in mouse epilepsy models and additional related gene family members, were selected. Most candidate genes tested were ion channels. Microsatellite markers within or very close to these genes were used to generate genotypes which were then subjected to either two-point linkage analysis or association chi-square studies. Using four different breeds, which may each have different forms of IE, increased the chances of potentially finding an associated gene in at least one of the breeds.

## Results

Call rates in each sample set for the microsatellite markers that were ultimately used in each analysis were approximately 90% or higher. Heterozygosity was recorded for each marker as a measure of that marker's extent of utility for this study. Average heterozygosity ± standard error of all markers analyzed for Beagles was 0.574 ± 0.024, for Vizslas was 0.569 ± 0.026, for ESS was 0.425 ± 0.030, and for GSMD was 0.341 ± 0.030. As expected, the average heterozygosity varied by breed and markers that were informative in one breed were not necessarily so in another breed. Out of all tested microsatellites, there were greater numbers of low heterozygosity (cut-off of 0.3) markers in the GSMD (27) and ESS (20) compared to the Vizslas (9) and Beagles (6), resulting in the lower average heterozygosities for the GSMD and ESS. For some genes, despite testing several microsatellites, results remain uninformative. For example, *GABRD *remains inconclusive in the ESS, despite testing three different microsatellites.

From the list of 52 candidate genes, 20 are known to be associated with inherited human epilepsy (indicated by a bolded entry with * in the tables) and two are associated with inherited mouse epilepsy models (indicated by a bolded entry with # in the tables). All 20 of the inherited human epilepsy-associated genes and both mouse model genes were analyzed at least once in all four of the test breeds, except for *CHRNA2 *in the Vizslas. However, not all of the other 30 genes were examined in every breed.

In the two-point linkage study, the two large Vizsla families were divided into ten smaller families to decrease complexity and inbreeding loops. The power of this ten family pedigree to detect significant linkage was demonstrated with simulated linkage analysis. The highest average LOD score for simulated linkage was 3.63, obtained by using a simulated marker with four alleles, no heterogeneity, and a recombination frequency of 0.001. The maximum attainable LOD score in these simulations was 8.46, obtained by using a simulated marker with three alleles, 5% heterogeneity, and a recombination frequency of 0.001. All of the simulation combinations obtained average LOD scores above 2.0 (suggestive linkage), demonstrating that the pedigrees are sufficient to detect linkage for a monogenic mutation. Results for the Vizslas are shown in Table 1. The majority of the candidate gene loci have very negative LOD scores and 43 genes were successfully excluded from being linked with IE in this breed. All of the human epilepsy-associated genes, with the exception of *CHRNA2*, and both of the mouse model genes were excluded. Four genes (*CACNA1I, CACNB3, CHRNA1*, and *SCN11A*) had low heterozygosity or monomorphism for the markers tested, therefore, these genes cannot be excluded. In addition, two genes (*CACNB1 *and *CACNB2*) had LOD values falling between -2.0 and 3.2, a grey zone of being neither linked nor unlinked; thus, conclusions cannot be drawn concerning possible linkage of these genes to IE in Vizslas. The most interesting of these was *CACNB1*, which had a LOD of 0.820 and a heterozygosity of 0.548.

**Table 1:** Vizsla two-point linkage analysis results

Gene Name	Designator	H	LOD at 0 cM (10 Fam)	Results
*ARX	First Set	0.555	(-)16.99	Excluded
*CACNA1A	First Set	0.163	(---)	Low Heterozygosity
*CACNA1A	Second Set	0.180	(---)	Low Heterozygosity
*CACNA1A	Third Set	0.142	(---)	Low Heterozygosity
*CACNA1A	Fourth Set	0.659	(-)8.06	Excluded
CACNA1B	First Set	0.757	(-)7.78	Excluded
CACNA1D	First Set	0.725	(-)16.18	Excluded
CACNA1E	First Set	0.783	(-)11.03	Excluded
CACNA1F	First Set	0.655	(-)23.95	Excluded
CACNA1G	First Set	0.584	(-)12.33	Excluded
*CACNA1H	First Set	0.000	(---)	Monomorphic
*CACNA1H	Second Set	0.423	(-)7.59	Excluded
CACNA1I	First Set	0.275	(---)	Low Heterozygosity
**#CACNA2D2**	First Set	0.495	(-)1.34	Neither Linked Nor Unlinked
**#CACNA2D2**	Second Set	0.665	(-)6.88	Excluded
CACNB1	First Set	0.548	0.82	Neither Linked Nor Unlinked
CACNB2	First Set	0.670	(-)1.39	Neither Linked Nor Unlinked
CACNB3	First Set	0.203	(---)	Low Heterozygosity
*CACNB4	First Set	0.781	(-)6.08	Excluded
**#CACNG2**	First Set	0.739	(-)21.61	Excluded
CACNG3	First Set	0.628	(-)10.69	Excluded
CACNG4	First Set	0.615	(-)8.70	Excluded
CACNG6	First Set	0.530	(-)8.35	Excluded
CHRNA1	First Set	0.291	(---)	Low Heterozygosity
CHRNA3	First Set	0.629	(-)3.58	Excluded
*CHRNA4 and KCNQ2	See KCNQ2			
CHRNA7	First Set	0.695	(-)9.65	Excluded
CHRNA9	First Set	0.420	(-)4.54	Excluded
CHRNA10	First Set	0.795	(-)6.43	Excluded
CHRNB1	First Set	0.587	(-)4.62	Excluded
*CHRNB2	First Set	0.725	(-)18.39	Excluded
CHRND	First Set	0.655	(-)5.05	Excluded
*CLCN2	First Set	0.665	(-)4.75	Excluded
DNM1	First Set	0.495	(-)3.63	Excluded
DNM1	Fourth Set	0.355	(-)3.77	Excluded
DNM1	Fifth Set	0.677	(-)3.93	Excluded
*GABRA1	First Set	0.605	(-)6.12	Excluded
GABRA2	First Set	0.575	(-)20.25	Excluded
GABRA6	First Set	0.646	(-)10.69	Excluded
*GABRD	First Set	0.745	(-)3.73	Excluded
*GABRG2	First Set	0.773	(-)22.33	Excluded
*GABRG2	Second Set	0.455	(-)4.39	Excluded
*KCNA1	First Set	0.716	(-)6.71	Excluded
KCNQ1	First Set	0.495	(-)3.10	Excluded
*KCNQ2 and CHRNA4	Second Set	0.578	(-)1.50	Neither Linked Nor Unlinked
*KCNQ2 and CHRNA4	Third Set	0.526	(-)0.49	Neither Linked Nor Unlinked
*KCNQ2 and CHRNA4	Fourth Set	0.676	(-)11.37	Excluded
*KCNQ3	First Set	0.711	(-)2.01	Excluded
KCNQ5	First Set	0.535	(-)5.29	Excluded
*LGI1	First Set	0.725	(-)9.89	Excluded
*ME2	First Set	0.625	(-)7.89	Excluded
*NHLRC1	Second Set	0.509	(-)8.49	Excluded
*SCN1A	First Set	0.600	(-)7.83	Excluded
*SCN1B	First Set	0.820	(-)8.52	Excluded
*SCN2A	First Set	0.833	(-)13.95	Excluded
SCN3A	First Set	0.000	(---)	Monomorphic
SCN3A	Second Set	0.545	(-)5.82	Excluded
SCN3B	First Set	0.751	(-)21.76	Excluded
SCN8A	First Set	0.805	(-)18.57	Excluded
SCN11A	First Set	0.255	(---)	Low Heterozygosity

Bolded genes marked with a * indicate those associated with human IE, and bolded genes marked with a # indicate those associated with mouse models of epilepsy. The breed cohort was assembled and statistics were performed as described in Materials and Methods. "Designator" indicates each set of primers, first, second, etc., designed for that gene and used in this breed (See Additional files 1 and 2). H = heterozygosity of the marker.

Results for the English Springer Spaniels are shown in Table 2. Pearson chi-square p-values are reported for this nonparametric association study. The majority of the loci (n = 36) had highly insignificant p-values, decreasing the likelihood that these genes are associated with IE in this breed; eighteen of the inherited human epilepsy genes were in this group, as were both of the mouse epilepsy genes. Nine genes, two of which are human epilepsy-associated genes (*GABRD *and *CHRNA2)*, had markers with heterozygosities falling below the 0.3 cut-off, prohibiting the drawing of conclusions about these genes. One gene, *GABRA1*, which is an inherited human epilepsy gene, initially resulted in marginally significant p-values for two different microsatellites: 0.074 (first set) and 0.033 (second set), however, a third microsatellite's alleles resulted in an insignificant p-value of 0.890. Additionally, when the Bonferroni correction was applied, these results were not significant; therefore, no further follow-up was initiated.

**Table 2:** English Springer Spaniel association chi-square results

Gene Name	Designator	H	Chi Square P-Value	Results
*ARX	First Set	0.485	0.843	Not Significant
*CACNA1A	First Set	0.312	0.308	Not Significant
CACNA1B	First Set	0.062	(---)	Low Heterozygosity
CACNA1D	First Set	0.000	(---)	Monomorphic
CACNA1E	First Set	0.684	0.314	Not Significant
CACNA1F	First Set	0.454	0.097	Not Significant
CACNA1G	First Set	0.467	0.916	Not Significant
*CACNA1H	First Set	0.734	0.644	Not Significant
CACNA1I	First Set	0.546	0.135	Not Significant
**#CACNA2D2**	First Set	0.369	0.898	Not Significant
CACNB1	First Set	0.734	0.744	Not Significant
CACNB2	First Set	0.619	0.305	Not Significant
CACNB3	First Set	0.362	0.113	Not Significant
*CACNB4	First Set	0.830	0.989	Not Significant
**#CACNG2**	First Set	0.413	0.963	Not Significant
CACNG3	First Set	0.653	0.130	Not Significant
CACNG4	First Set	0.635	0.220	Not Significant
CACNG6	First Set	0.561	0.267	Not Significant
CHRNA1	First Set	0.538	0.249	Not Significant
*CHRNA2	First Set	0.233	(---)	Low Heterozygosity
*CHRNA4 and KCNQ2	See KCNQ2			
CHRNA5	First Set	0.436	0.788	Not Significant
CHRNA7	First Set	0.692	0.622	Not Significant
CHRNA9	First Set	0.259	(---)	Low Heterozygosity
CHRNB1	First Set	0.207	(---)	Low Heterozygosity
*CHRNB2	First Set	0.173	(---)	Low Heterozygosity
*CHRNB2	Second Set	0.198	(---)	Low Heterozygosity
*CHRNB2	Third Set	0.321	0.982	Not Significant
*CLCN2	First Set	0.492	0.719	Not Significant
DNM1	First Set	0.227	(---)	Low Heterozygosity
DNM1	Second Set	0.528	0.886	Not Significant
DNM1	Third Set	0.700	0.598	Not Significant
DNM1	Fourth Set	0.714	0.875	Not Significant
DNM1	Fifth Set	0.618	0.853	Not Significant
*GABRA1	First Set	0.416	0.074	Inconclusive
*GABRA1	Second Set	0.490	0.033	Inconclusive
*GABRA1	Third Set	0.369	0.890	Not Significant
*GABRD	First Set	0.257	(---)	Low Heterozygosity
*GABRD	Second Set	0.223	(---)	Low Heterozygosity
*GABRD	Third Set	0.295	(---)	Low Heterozygosity
*GABRG2	First Set	0.663	0.443	Not Significant
*KCNA1	First Set	0.587	0.965	Not Significant
KCND2	First Set	0.233	(---)	Low Heterozygosity
*KCNQ2 and CHRNA4	Second Set	0.738	0.710	Not Significant
*KCNQ3	First Set	0.541	0.474	Not Significant
KCNQ5	First Set	0.206	(---)	Low Heterozygosity
*LGI1	First Set	0.641	0.834	Not Significant
*ME2	First Set	0.492	0.619	Not Significant
*NHLRC1	Second Set	0.684	0.784	Not Significant
*SCN1A	First Set	0.199	(---)	Low Heterozygosity
*SCN1A	Second Set	0.082	(---)	Low Heterozygosity
*SCN1A	Third Set	0.211	(---)	Low Heterozygosity
*SCN1A	Fourth Set	0.337	0.968	Not Significant
*SCN1B	First Set	0.676	0.216	Not Significant
*SCN2A	First Set	0.766	0.670	Not Significant
SCN3A	First Set	0.000	(---)	Monomorphic
SCN3A	Second Set	0.069	(---)	Low Heterozygosity
SCN3B	First Set	0.563	0.834	Not Significant
SCN8A	First Set	0.070	(---)	Low Heterozygosity
SCN8A	Second Set	0.000	(--)	Monomorphic
SCN11A	First Set	0.637	0.396	Not Significant

Bolded genes marked with a * indicate those associated with human IE, and bolded genes marked with a # indicate those associated with mouse models of epilepsy. The breed cohort was assembled and statistics were performed as described in Materials and Methods. "Designator" indicates each set of primers, first, second, etc., designed for that gene and used in this breed (See Additional files 1 and 2). H = heterozygosity of the marker.

Results for the Greater Swiss Mountain Dogs are shown in Table 3. As with the ESS, Pearson chi-square p-values are reported for this association study. Again, the majority of the loci (n = 35) had highly insignificant p-values and are unlikely to be associated with IE in the GSMD; this included 17 of the 20 inherited human epilepsy-associated genes and both of the mouse model genes. This breed had the lowest overall average heterozygosity and results for eleven genes, three of which are human epilepsy-associated (*GABRD*, *KCNA1*, and *ME2*), could not be determined due to low heterozygosity. The first marker analyzed for *KCND2 *resulted in a p-value of 0.036, prompting the examination of two additional markers. One of these was uninformative, but the other had a heterozygosity of 0.729 and a p-value of 0.155, rendering this gene not significant and unlikely to be associated to IE in GSMD. Additionally, *CHRNB2*, a gene that is associated with human epilepsy, resulted in a p-value of 0.055. While this p-value is potentially suggestive of association, when correcting for multiple testing with the Bonferroni correction, it does not approach significance.

**Table 3:** Greater Swiss Mountain Dog association chi-square results

Gene Name	Designator	H	Chi Square P-Value	Results
*ARX	First Set	0.396	0.606	Not Significant
*CACNA1A	First Set	0.000	(---)	Monomorphic
*CACNA1A	Second Set	0.408	0.740	Not Significant
CACNA1B	First Set	0.370	0.905	Not Significant
CACNA1D	First Set	0.699	0.529	Not Significant
CACNA1E	First Set	0.675	0.953	Not Significant
CACNA1F	First Set	0.106	(---)	Low Heterozygosity
CACNA1G	First Set	0.000	(---)	Monomorphic
CACNA1G	Second Set	0.023	(---)	Low Heterozygosity
*CACNA1H	First Set	0.000	(---)	Monomorphic
*CACNA1H	Second Set	0.517	0.272	Not Significant
CACNA1I	First Set	0.483	0.449	Not Significant
**#CACNA2D2**	First Set	0.308	0.442	Not Significant
CACNB1	First Set	0.261	(---)	Low Heterozygosity
CACNB2	First Set	0.000	(---)	Monomorphic
CACNB3	First Set	0.322	0.718	Not Significant
*CACNB4	First Set	0.628	0.709	Not Significant
**#CACNG2**	First Set	0.690	0.382	Not Significant
CACNG3	First Set	0.000	(---)	Monomorphic
CACNG3	Second Set	0.591	0.527	Not Significant
CACNG4	First Set	0.298	(---)	Low Heterozygosity
CACNG6	First Set	0.482	0.323	Not Significant
CHRNA1	First Set	0.491	0.730	Not Significant
CHRNA1	Second Set	0.670	0.256	Not Significant
CHRNA1	Third Set	0.656	0.374	Not Significant
CHRNA1	Fourth Set	0.452	0.582	Not Significant
*CHRNA2	First Set	0.461	0.644	Not Significant
*CHRNA4 and KCNQ2	See KCNQ2			
CHRNA5	First Set	0.389	0.483	Not Significant
CHRNA7	First Set	0.810	0.924	Not Significant
CHRNA9	First Set	0.615	0.875	Not Significant
CHRNB1	First Set	0.000	(---)	Monomorphic
*CHRNB2	First Set	0.333	0.055	Not Significant
*CLCN2	First Set	0.519	0.600	Not Significant
DNM1	First Set	0.139	(---)	Low Heterozygosity
DNM1	Second Set	0.364	0.799	Not Significant
DNM1	Third Set	0.500	0.834	Not Significant
DNM1	Fourth Set	0.000	(---)	Monomorphic
DNM1	Fifth Set	0.484	0.464	Not Significant
*GABRA1	First Set	0.549	0.354	Not Significant
*GABRD	First Set	0.088	(---)	Low Heterozygosity
*GABRD	Second Set	0.088	(---)	Low Heterozygosity
*GABRD	Third Set	0.081	(---)	Low Heterozygosity
*GABRG2	First Set	0.219	(---)	Low Heterozygosity
*GABRG2	Second Set	0.458	0.929	Not Significant
*KCNA1	First Set	0.171	(---)	Low Heterozygosity
KCND2	First Set	0.480	0.036	Inconclusive
KCND2	Second Set	0.729	0.155	Not Significant
KCND2	Third Set	0.124	(---)	Low Heterozygosity
*KCNQ2 and CHRNA4	First Set	0.441	0.677	Not Significant
*KCNQ3	First Set	0.382	0.904	Not Significant
KCNQ5	First Set	0.100	(---)	Low Heterozygosity
*LGI1	First Set	0.440	0.244	Not Significant
*ME2	First Set	0.219	(---)	Low Heterozygosity
*NHLRC1	Second Set	0.405	0.240	Not Significant
*SCN1A	First Set	0.749	0.706	Not Significant
*SCN1B	First Set	0.000	(---)	Monomorphic
*SCN1B	Second Set	0.000	(---)	Monomorphic
*SCN1B	Third Set	0.026	(---)	Low Heterozygosity
*SCN1B	Fourth Set	0.485	0.233	Not Significant
*SCN2A	First Set	0.686	0.767	Not Significant
SCN3A	First Set	0.000	(---)	Monomorphic
SCN3A	Second Set	0.437	0.378	Not Significant
SCN3B	First Set	0.000	(---)	Monomorphic
SCN3B	Second Set	0.000	(---)	Monomorphic
SCN8A	First Set	0.152	(---)	Low Heterozygosity
SCN8A	Second Set	0.576	0.996	Not Significant
SCN11A	First Set	0.597	0.967	Not Significant

Bolded genes marked with a * indicate those associated with human IE, and bolded genes marked with a # indicate those associated with mouse models of epilepsy. The breed cohort was assembled and statistics were performed as described in Materials and Methods. "Designator" indicates each set of primers, first, second, etc., designed for that gene and used in this breed (See Additional files 1 and 2). H = heterozygosity of the marker.

Results for the Beagles are shown in Table 4. In this breed association study, highly insignificant p-values for 43 candidate genes indicate that these genes are likely not associated with IE in Beagles. All twenty of the inherited human epilepsy-associated genes and both of the mouse model genes had insignificant p-values in this breed. Only three genes (*CACNA1D*, *CACNG3*, and *KCNQ5*) were inconclusive due to low heterozygosity in this breed. *CHRNA1 *gave a p-value of 0.05 on its initial marker, leading to the development of primers for additional microsatellites. Two of these, *CHRNA1*'s third set and fourth set of primers, gave p-values of 0.072 and 0.056, respectively, but *CHRNA1*'s second set of primers resulted in a highly insignificant p-value of 0.600, making it unlikely that this locus is associated with IE in this breed. Two other loci, both human epilepsy-associated genes, though insignificant, had lower p-values: *KCNQ3 *at 0.077 and *LGI1 *at 0.070. Again, applying correction for multiple testing indicates that these p-values are not significant.

**Table 4:** Beagle association chi-square results

Gene Name	Designator	H	Chi Square P-Value	Results
*ARX	First Set	0.508	0.643	Not Significant
*CACNA1A	First Set	0.636	0.813	Not Significant
CACNA1B	First Set	0.836	0.765	Not Significant
CACNA1D	First Set	0.123	(---)	Low Heterozygosity
CACNA1E	First Set	0.697	0.254	Not Significant
CACNA1F	First Set	0.671	0.578	Not Significant
CACNA1G	First Set	0.640	0.092	Not Significant
*CACNA1H	Second Set	0.344	0.873	Not Significant
CACNA1I	First Set	0.570	0.365	Not Significant
**#CACNA2D2**	First Set	0.438	0.917	Not Significant
CACNB1	First Set	0.318	0.661	Not Significant
CACNB2	First Set	0.612	0.577	Not Significant
CACNB3	First Set	0.577	0.148	Not Significant
*CACNB4	First Set	0.864	0.753	Not Significant
**#CACNG2**	First Set	0.720	0.502	Not Significant
CACNG3	First Set	0.135	(---)	Low Heterozygosity
CACNG4	First Set	0.633	0.816	Not Significant
CACNG6	First Set	0.505	0.413	Not Significant
CHRNA1	First Set	0.686	0.050	Not Significant
CHRNA1	Second Set	0.506	0.600	Not Significant
CHRNA1	Third Set	0.661	0.072	Not Significant
CHRNA1	Fourth Set	0.790	0.056	Not Significant
*CHRNA2	First Set	0.664	0.590	Not Significant
*CHRNA4 and KCNQ2	See KCNQ2			
CHRNA7	First Set	0.741	0.513	Not Significant
CHRNA9	First Set	0.665	0.694	Not Significant
CHRNB1	First Set	0.491	0.555	Not Significant
*CHRNB2	First Set	0.583	0.885	Not Significant
*CLCN2	First Set	0.762	0.577	Not Significant
DNM1	First Set	0.278	(---)	Low Heterozygosity
DNM1	Second Set	0.386	0.693	Not Significant
DNM1	Third Set	0.588	0.314	Not Significant
DNM1	Fourth Set	0.565	0.953	Not Significant
DNM1	Fifth Set	0.563	0.430	Not Significant
*GABRA1	First Set	0.523	0.345	Not Significant
GABRA2	First Set	0.502	0.265	Not Significant
GABRA6	First Set	0.575	0.266	Not Significant
*GABRD	First Set	0.684	0.554	Not Significant
*GABRG2	First Set	0.748	0.292	Not Significant
*GABRG2	Second Set	0.447	0.849	Not Significant
*KCNA1	First Set	0.758	0.356	Not Significant
KCND2	First Set	0.530	0.919	Not Significant
KCND2	Second Set	0.706	0.698	Not Significant
KCND2	Third Set	0.728	0.455	Not Significant
*KCNQ2 and CHRNA4	Second Set	0.805	0.627	Not Significant
*KCNQ3	First Set	0.655	0.077	Not Significant
KCNQ5	First Set	0.106	(---)	Low Heterozygosity
*LGI1	First Set	0.234	(---)	Low Heterozygosity
*LGI1	Second Set	0.708	0.070	Not Significant
*ME2	First Set	0.585	0.282	Not Significant
*NHLRC1	First Set	0.605	0.739	Not Significant
*SCN1A	First Set	0.118	(---)	Low Heterozygosity
*SCN1A	Third Set	0.328	0.551	Not Significant
*SCN1B	First Set	0.679	0.425	Not Significant
*SCN1B	Second Set	0.649	0.885	Not Significant
*SCN2A	First Set	0.680	0.763	Not Significant
SCN3B	First Set	0.750	0.127	Not Significant
SCN8A	First Set	0.730	0.110	Not Significant
SCN11A	First Set	0.689	0.572	Not Significant

Bolded genes marked with a * indicate those associated with human IE, and bolded genes marked with a # indicate those associated with mouse models of epilepsy. The breed cohort was assembled and statistics were performed as described in Materials and Methods. "Designator" indicates each set of primers, first, second, etc., designed for that gene and used in this breed (See Additional files 1 and 2). H = heterozygosity of the marker.

## Discussion

This study was designed to use LD to exploit a possible founder effect suspected to be responsible for IE in these breeds by testing as many candidate genes as possible using microsatellite markers. Microsatellites have been shown to be a practical and useful tool in candidate gene studies [29]. Marker locations were selected within average blocks of canine LD so that one marker's result could, in theory, accurately represent the entire gene. The use of multiple breeds allowed for the possibility of assessing whether a discovered mutation was unique to one breed, suggesting the suspected founder effect specific to that breed, or was observed in several breeds, indicating a much older mutation. While no mutations were identified to draw definitive conclusions, it is not unreasonable to speculate that in dogs the underlying genetic basis of IE may vary by breed, as it is known that the rare human monogenic IEs are typically isolated by family [30-35]. These experiments incorporated both linkage analysis and association analysis, each having different strengths. While linkage analysis is the more powerful of these two statistical analysis methods for detecting Mendelian disease mutations, it requires samples from appropriate family structures. Conversely, association analysis does not require family-based samples, and it is generally considered more powerful than linkage analysis in detecting polygenic disease associations. Sequencing of the subset of human IE-associated candidate genes was not pursued for this study, as brain tissues for obtaining cDNAs for most cost efficient sequencing were not available.

Overall marker informativeness was highly influenced by sample population. The Vizslas and Beagles had excellent average heterozygosities (0.569 and 0.574, respectively), while the ESS and GSMD were much lower (0.425 and 0.341, respectively). This may be due, in part, to the sample cohorts: the ESS and GSMD were set up as discordant full- or half-sibling pairs, whereas the Vizslas were constructed as large pedigrees, and the Beagles were case/control matched pairs that did not share common ancestors to at least the grandparent level. Reduced marker heterozygosity across breeds could also be influenced by a higher degree of inbreeding within the ESS and GSMD sample cohorts in this study, and the lower average heterozygosities of these two breeds may suggest a founder effect that could eventually help uncover associations. However, marker informativeness is not entirely dependent on sample population, as evidenced by *CACNA1A*, which was polymorphic enough to be confidently insignificant after analyzing one or two microsatellites in ESS, GSMD, and Beagles, but remained inconclusive through three markers in the Vizslas and ultimately required a fourth microsatellite to achieve a suitable heterozygosity in this breed. The accepted marker heterozygosity of 0.3 is perhaps low, especially when analyzing only a single microsatellite for each candidate gene, however, for many of the insignificant microsatellite results in all four breeds the heterozygosity was > 0.5.

Ultimately, 16 of the 20 human epilepsy-associated candidate genes (*ARX*, *CACNA1A*, *CACNA1H*, *CACNB4*, *CHRNA4*, *CHRNB2*, *CLCN2*, *GABRA1*, *GABRG2*, *KCNQ2*, *KCNQ3*, *LGI1*, *NHLRC1*, *SCN1A*, *SCN1B*, and *SCN2A*) and both of the mouse model genes (*CACNA2D2 *and *CACNG2*) were either excluded (with linkage) or demonstrated insignificant association to IE in all four of the breed cohorts. Another two of them (*KCNA1 *and *ME2*) were insignificant in three out of four breed cohorts, remaining inconclusive only in the GSMD. One gene, *GABRD*, was inconclusive in two breeds (ESS and GSMD), and *CHRNA2 *was inconclusive in ESS and was not tested in Vizslas. Insignificant association to IE was demonstrated in most of the additional candidate genes tested. For every breed, however, there were a handful of markers that were inconclusive, due to low heterozygosity.

The few markers with potentially interesting results (uncorrected p-values of < 0.05) that were not excluded by follow-up markers, including *CACNB1 *in the Vizsla, *CHRNB2 *in the GSMD, and *KCNQ3 *and *LGI1 *in the Beagle, were of minimal interest when correcting for multiple testing. With the Bonferroni correction, a p-value of 0.05 considered as statistically significant would be lowered to 0.0025 for twenty tests, and further lowered to 0.00125 for forty tests; p-values of this magnitude were not obtained for any marker, and all breeds were tested on more than forty genes. The potential of false positives due to population stratification must also be considered for these few genes with suggestive results in the association studies, despite attempts to control for the degree of relatedness. Haplotype association analysis was not possible with this data since there were not enough closely-spaced markers to generate haplotypes. Conclusive confirmation or exclusion of these loci can be performed in the future with newer technologies such as whole-genome SNP arrays, which can generate vast amounts of data in less time.

It is possible that the populations tested were underpowered to detect association if it existed. Utilizing a sibling-pair case-control design could decrease power because the control dog may also carry the risk allele. However, this situation is difficult to avoid in highly inbred dog populations, and the sibling-pair case-control design should aid in avoiding population stratification, which can create false positive results. Although sample size requirements for canine association studies have not been precisely defined, based on average linkage disequilibrium estimates in dog breeds, a starting point of approximately 25 cases and 25 controls appears to be adequate to find statistical significance for a completely penetrant recessive trait [36]. The number increases to approximately 50 cases and 50 controls when a trait is dominant, and for more complex traits as many as 100 dogs in each group may be necessary. Therefore, it is possible that there were not adequate numbers of dogs in the association studies, particularly if IE is not monogenic. A meta-analysis might improve power by pooling data from the three association study breeds (ESS, GSMD, and Beagle). However, this proved to be impractical because 1) many microsatellites had varying informativeness in each breed, so that pooling data would only work for a single microsatellite that was informative for a gene in all three breeds and 2) microsatellites by their very nature are highly polymorphic and there was often little overlap in alleles between breeds. Lastly, if IE has a different genetic basis in each of these breeds, pooling data across breeds seems unlikely to yield interesting results.

In the linkage analysis of Vizsla pedigrees, it is possible to conclude that these candidate gene loci are truly excluded, due to convincingly negative LOD scores at zero centiMorgans. However, if the inheritance and age dependent penetrance assumptions for the Vizsla model are incorrect, then the present linkage study is flawed. These input assumptions were based on previous study of IE in this breed [23] and on the best information available. Conversely, in the association studies of the other three breeds, it is not possible to specifically state that the loci are "excluded", as there is some degree of doubt that one microsatellite marker is adequate to provide convincing evidence for exclusion of a locus with this type of study. The addition of more markers would help to verify these insignificant results and truly exclude a locus. That work is beyond the scope of this study and is better addressed with whole-genome SNP analysis. The latest commercial canine SNP array, with over 170,000 SNPs, became available well after these studies were initiated. Further, the excluded and insignificant genes reported in the present study may only reflect the specific breeds examined, or even lines within these breeds, and results should not necessarily be extrapolated to other breeds.

It is possible that canine IE, like the majority of human IE, is a genetically complex disease in most breeds and that multiple loci contribute to susceptibility in any given breed. The present study would very likely have detected a major contributing locus, and it is unlikely that a truly causative locus is being excluded as a false negative. In humans, the vast majority of IE remains genetically unexplained and is considered to be polygenic [37-39]. A recent study by Oberbauer et al. [40] utilized microsatellites in a genome-wide linkage scan for epilepsy loci in the Belgian shepherd dog and concluded that the disease was highly polygenic, reporting a tentative six QTLs. These results further support the conclusion that canine IE is a more complex disease than originally hypothesized. Whole-genome association analyses with SNP arrays are likely the platform of choice for further studies of IE, as they can query tens of thousands of markers simultaneously across the genome and are better able to identify multiple susceptibility loci. Additionally, copy number variants (CNVs) have been increasingly shown to be involved in neurologic disorders such as autism [41,42] and schizophrenia [43,44], as well as epilepsy [45]. CNV studies of canine epilepsy may reveal this as a similar mechanism for disease in both species.

Ultimately, canine IE found to be significantly associated with DNA markers or with a mutation in a specific gene would allow genetic tests to be developed to assist dog breeders with decreasing the incidence of this disease. This would be most effective for monogenic, highly penetrant forms of IE, but if the disease proves to be genetically complex, with multiple genes contributing and less than 100% penetrance, then such a test could still possibly be used to provide a relative risk for IE development within a breed. In any event, discovery of IE associated gene loci in dogs may not only improve the understanding of canine health, but could advance the study of neurobiology and human health as well.

## Conclusions

The present study did not identify any obvious association or linkage between IE and the microsatellite markers tested for the various candidate genes in any of the four dog breeds. It is possible that IE may in fact be a polygenic disorder even in highly inbred dog breeds, which underscores the challenging nature of investigating inherited epilepsy. Eventually identifying the genetic causes for IE in dogs would have a significant impact on canine health, as well as providing a useful model for IE in humans.

## Methods

### Case Definition

Since seizure activity can result from numerous discernable abnormalities, and only dogs very likely to be affected with IE were to be included in the study, the case definition was designed to eliminate, as much as possible, all other likely causes of seizures. Therefore, in order to qualify for "case" status for IE, each affected dog must have had two or more seizures with clinical normalcy between episodes, a veterinarian-administered normal physical examination and neurological examination, as well as normal bloodwork, including a complete blood count and blood chemistry profile, and, when possible, a bile acids assay. Since IE has been shown to be statistically much more probable when the dog is aged 1 to 5 years at the time of its first seizure [46], only dogs whose first seizure occurred during this time frame were included. If seizures started prior to 1 year of age or after 5 years of age, extra criteria were then required to enroll a dog, including a normal MRI or CT, and/or normal CSF analysis. EEG was not used as a case criterion due to its limited availability in clinical veterinary practice. To be classified as unaffected, the dog must have reached at least five years of age and be completely free of known seizure activity. In the situation where a case was identified at a young age, and its full- or half-sibling was used as a control, the dogs were followed for years until the unaffected dog had reached at least five years of age.

Dogs in these studies were privately owned and voluntarily enrolled by their owners with informed consent. Cases were solicited through breed publications, presentations at local and national breed club meetings, and word of mouth. For each enrolled dog, a general health survey, a signed owner consent form, and up to ten mLs of whole blood in an EDTA tube were required. Blood samples were obtained via venipuncture either at the dog's regular veterinarian or at specially-organized blood-draws at breed shows across the USA. This study was approved by the University of Minnesota IACUC.

Owners of dogs affected with seizures were asked to fill out an additional questionnaire which asked for information about the dog's seizure characteristics, preictal and postictal signs, previous medical history, and any anti-epileptic drug therapy; these were submitted by phone, fax, postal mail or e-mail. Any additional follow-up questions were conducted via telephone interview. Finally, medical records for each affected dog were obtained from the dog's regular veterinarian and any specialists (if applicable); these were inspected for bloodwork results and the veterinarian's physical and neurological examination findings. Dogs were assigned case, control, or unknown status after careful evaluation of this information. "Unknown" status was reserved for those dogs displaying suspicious seizure or seizure-like activity, but couldn't be classified as a case for various reasons; for example, if the dog had only one recorded seizure or had suffered head trauma prior to onset of the first seizure.

### DNA Isolation

DNA was isolated from whole blood following standard protocols, utilizing the Puregene DNA Isolation Kit from Qiagen Inc. (Valencia, CA).

### Breed Cohorts

Cohorts of affected and unaffected dogs for genotyping were assembled for each of the four test breeds - the Vizsla, the GSMD, the ESS, and the Beagle. Ultimately, the available samples determined what type of genetic analysis was conducted for each breed.

### Vizslas

**Figure 1:**
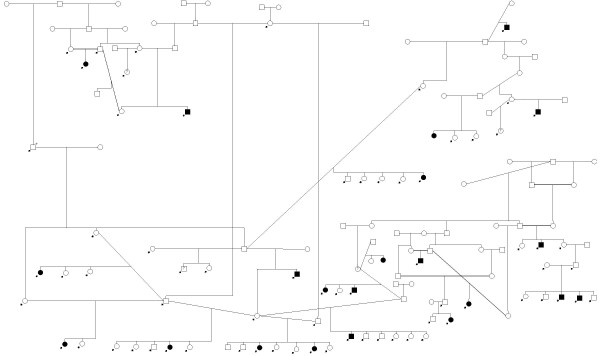
**Vizsla linkage family 1 pedigree**. Pedigree of Vizsla family 1. Squares and circles represent males and females, respectively. Filled shapes represent cases; those with question marks represent unknown phenotype status. Dogs with arrows were genotyped in this study. The dog marked with an arrow and a + is included on both families 1 & 2, effectively making this one very large family. The Vizsla pedigrees were broken into ten smaller families to decrease inbreeding loops before being analyzed in linkage analysis. Three dogs representing one of the ten sub-families are not shown on either pedigree.

**Figure 2:**
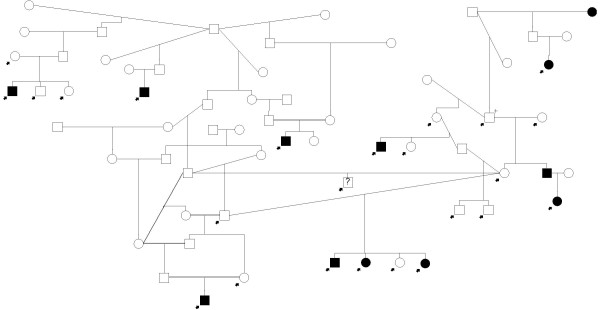
**Vizsla linkage family 2 pedigree**. Pedigree of Vizsla family 2. Squares and circles represent males and females, respectively. Filled shapes represent cases; those with question marks represent unknown phenotype status. Dogs with arrows were genotyped in this study. The dog marked with an arrow and a + is included on both families 1 & 2, effectively making this one very large family. The Vizsla pedigrees were broken into ten smaller families to decrease inbreeding loops before being analyzed in linkage analysis. Three dogs representing one of the ten sub-families are not shown on either pedigree.

For Vizslas, the depth of the available DNA samples from multiple siblings, parents, and many grandparents allowed the development of multi-generation pedigrees (Figure 1 and Figure 2) suitable for genetic linkage analysis. 96 dogs were included: 31 affected dogs, 60 unaffected dogs, and 5 dogs with unknown phenotype status. These dogs were separated into two large family pedigrees shown in Figures 1 and 2, and were subsequently broken into ten smaller families to decrease inbreeding loops for linkage analysis and calculation of LOD scores.

### Greater Swiss Mountain Dogs

For the GSMD, discordant full-sibling or half-sibling pairs of affected (n = 24) and unaffected (n = 24) dogs were assembled and used in association studies. Siblings were used to help avoid spurious association from population stratification. In a few instances, two different pairs of siblings were related to each other, such as sharing one parent.

### English Springer Spaniels

Similar to the GSMD, the ESS were assembled into discordant full- or half-sibling pairs of affected (n = 24) and unaffected (n = 24) dogs and were used in association studies. As in the GSMD, occasionally the pairs of siblings were also related to one another; this was impossible to avoid due to the inbred nature of the breed.

### Beagles

Association studies were also conducted for the Beagles. Due to lack of available sibling pairs, pedigree analyses were used to ensure that only dogs with no common grandparents were utilized. This strategy has previously shown to be adequate for canine genetic mutation detection [47]. Twenty-four affected dogs and 24 unaffected dogs were selected for this dataset.

### Candidate Gene Selection

A non-exhaustive list of candidate genes known to be involved with inherited human epilepsy was assembled, including potassium channels *KCNA1*, *KCNQ2*, and *KCNQ3*; calcium channels *CACNA1A*, *CACNA1H*, and *CACNB4*; sodium channels *SCN1A*, *SCN2A*, and *SCN1B*; GABA receptors *GABRA1*, *GABRD*, and *GABRG2*; acetylcholine receptors *CHRNA2*, *CHRNA4*, and *CHRNB2*; and chloride channel *CLCN2*; as well as novel (ie, non-ion channel and non-neurotransmitter receptor) genes malic enzyme 2 (*ME2*), leucine-rich glioma inactivated 1 (*LGI1*), NHL repeat containing 1 (*NHLRC1*), and aristaless related homeobox (*ARX*), for a total of 20 human epilepsy-associated genes [30,48-54]. Two candidate genes associated with mouse epilepsy models were also included: *CACNA2D2*, (the ducky mouse) [55] and *CACNG2 *(the stargazer mouse) [56].

In addition to this core set of genes, further genes were selected from the same gene families based on their presence in neural tissue, including: calcium channels *CACNA1B*, *CACNA1D, CACNA1E, CACNA1F, CACNA1G, CACNA1I, CACNB1, CACNB2, CACNB3, CACNG3, CACNG4*, and *CACNG6*; sodium channels *SCN3A, SCN3B*, *SCN8A*, and *SCN11A*; GABA receptors *GABRA2*, and *GABRA6*; acetylcholine receptors *CHRNA1, CHRNA3, CHRNA5*, *CHRNA9, CHRNA10, CHRNB1*, and *CHRND *as well as *CHRNA7*, which has had suggested linkage with human IE [57]; and potassium channels *KCND2, KCNQ1*, and *KCNQ5*. Finally, the dynamin-1 gene (*DNM1) *was also examined. This gene was recently found to be mutated in the canine disease termed exercise induced collapse [58] and a mutation in dynamin-1 has recently been characterized in the fitful mouse model, wherein heterozygotes for the mutation experience recurrent seizures and homozygotes for the mutation often have lethal seizures and other neurosensory deficits [59]. Therefore, a total of 52 candidate genes were chosen for this study.

### Primer design, PCR, and Genotyping

Human mRNA sequence was obtained for each gene from the National Center for Biotechnology Information [60]. This sequence was BLATed on the University of California - Santa Cruz Genome Browser website [61] against the May 2005 dog genome assembly to identify the resultant genomic position of each gene in the dog. Microsatellites were selected either within the gene or closely flanking the gene utilizing the Variation and Repeat - Microsatellite feature of this browser. After capturing the microsatellite's flanking sequence, primer pairs to amplify each microsatellite were designed using Primer3 [62]. An additional 5' tail sequence was added to each right (reverse) primer [63] to generate fluorescent labels in PCR. All primer sequences are shown in Additional file 1, and Additional file 2 lists the Mb positions of each gene and microsatellite. Every attempt was made to select microsatellites less than 1 Mb distant from the gene, in order to remain within estimated blocks of canine linkage disequilibrium (LD) [36] for each gene. LD is the non-random association of alleles at two loci and, within dog breeds, can extend over distances of several megabases [36]. However, for many of the candidate genes, several microsatellites (and therefore several sets of primers) needed to be tested due to the microsatellite's lack of informativeness (monomorphism or low heterozygosity), and eventually the only useable microsatellite was located further away. Additional file 2 indicates which microsatellites (n = 3) were >1 Mb distant. *CHRNA4 *and *KCNQ2 *were close enough to one another in the canine genome that one marker was used for both genes.

Each microsatellite was PCR amplified in 15-μL reactions including 12.5 ng canine DNA, 10X PCR Buffer, 2.5 pmol of the forward primer and 0.75 pmol of the reverse-tailed primer, 0.78 pmol of a fluorescent dye-labeled primer complementary to the tail sequence, 20 μM dNTP mix, and 0.5 units of *Taq *DNA polymerase (Qiagen Hot StarTaq, Qiagen Inc, Valencia, CA), with dH_2_O to final volume. PCR cycling conditions were 95°C for 20 minutes, then 40 cycles of the following: 94°C for 30 seconds, 58°C for 30 seconds, and 72°C for 30 to 60 seconds (depending on product size), and a final extension of 72°C for 15 minutes. PCR products were prepared for capillary gel electrophoresis according to manufacturer's recommendations and size separated using the Beckman CEQ 8000 automated DNA-fragment analyzer with fluorescence detection (Beckman Coulter Inc, Fullerton CA). Genotype data were analyzed with instrument software, and alleles were manually verified.

### Statistical Analysis

Marker heterozygosity was calculated using the following formula: *H *= 1 - Σ*p_i_*^2^, where *p_i _*is the population frequency of the *i*th allele, and *H *is the probability that a random individual is heterozygous for any two alleles at a gene locus with allele frequencies, *p_i _*[64]. A cut-off of 0.3 was selected for heterozygosity; markers with heterozygosity falling below this cut-off were not considered informative enough to confidently claim a non-significant result for a gene, since less than one third of all individuals would be expected to be heterozygous at that locus [64].

In order to determine the power of the ten Vizsla family pedigrees to detect linkage, simulated linkage was performed with the FASTSLINK program [65-67]. Multiple parameters were used, including combinations of 3 and 4 equally frequent alleles, 0 and 5% heterogeneity (proportion of unlinked families) and recombination frequencies of 0.01 and 0.001. Recombination frequencies were kept very small since the microsatellites tested were within or very near to the candidate genes. Two hundred simulations were performed for each set of parameters, assuming autosomal recessive inheritance.

Parametric two-point linkage analysis was utilized for Vizsla marker data, with significant evidence for linkage a LOD (logarithm of odds) score of 3.2 or greater [68]. A LOD score of less than -2.0 was considered to exclude a locus. Input assumptions were an autosomal recessive mode of inheritance with 95% penetrance for the disease genotype, and a disease allele frequency of 0.20; these assumptions were based on previously published data concerning IE in this breed [23]. Two-point LOD scores were calculated using the FASTLINK version of MLINK [69-72].

For the GSMD, ESS, and Beagles, the allele frequencies were subjected to a nonparametric chi-square test for independence (association analysis) [73]. Alleles with frequencies of less than 10% (minor alleles) were removed before conducting the chi-square analysis. A Pearson chi-square p-value of 0.05 was first used as the level of significance necessary to detect significantly different allele frequencies between affected and unaffected dogs. Subsequently, a Bonferroni corrected p-value of 0.001, correcting for ~52 tests, was considered evidence for strong association. Power analyses were not specifically conducted for these three breeds, as sample size requirements for canine association studies have not been precisely defined.

## Authors' contributions

KJE participated in conceiving the study, carried out the methods including choosing microsatellites, designing primers, genotyping, statistical analyses, and drafted the manuscript. EEP conceived the study and study design, and coordinated sample accession. KMM participated in carrying out methods including constructing pedigrees and genotyping, as well as sample accession. JRM conceived the study and study design and contributed significant manuscript review. All authors read and approved the final manuscript.

## Supplementary Material

Additional file 1 **Primer sequences for candidate gene microsatellites**. Sequence information and product sizes are presented for primers designed for microsatellites examined for each candidate gene. Bolded genes marked with a * indicate those associated with human epilepsy, and bolded genes marked with a # indicate those associated with mouse models of epilepsy. Two of the DNM1 marker primer pairs, DNM1 Third Set and DNM1 Fifth Set, are taken from the University of California-Davis Canine Genetic Linkage Map [74], and are named therein 0945 and 0946, respectively. CFA = canis familiaris chromosome. Click here for file

Additional file 2 **Candidate gene locations and primer locations**. All pertinent canine chromosomal location information is presented for each gene and all microsatellites examined for that gene. Bolded genes marked with a * indicate those associated with human epilepsy, and bolded genes marked with a # indicate those associated with mouse models of epilepsy. Two of the DNM1 marker primer pairs, DNM1 Third Set and DNM1 Fifth Set, are taken from the University of California-Davis Canine Genetic Linkage Map [74], and are named therein 0945 and 0946, respectively. ^ Bolded entries in this column indicate microsatellites located outside the gene itself and further than 1 Mb away (n = 3). Mb positions given for microsatellites are the first nucleotide of each microsatellite. Mb positions were taken from Ensembl [75] and the UCSC Genome Browser [61]. CFA = canis familiaris chromosome. Click here for file
