# Supplementary material for: Candidate genes for idiopathic epilepsy in four dog breeds
Source: BMC Genet. 2011 Apr 25;12:38. doi: 10.1186/1471-2156-12-38 (PMC3111397; doi:10.1186/1471-2156-12-38)
Supplement: Additional file 1 — Primer sequences for candidate gene microsatellites. Sequence information and product sizes are presented for primers designed for microsatellites examined for each candidate gene. Bolded genes marked with a * indicate those associated with human epilepsy, and bolded genes marked with a # indicate those associated with mouse models of epilepsy. Two of the DNM1 marker primer pairs, DNM1 Third Set and DNM1 Fifth Set, are taken from the University of California-Davis Canine Genetic Linkage Map [74], and are named therein 0945 and 0946, respectively. CFA = canis familiaris chromosome. [file 1471-2156-12-38-S1.PDF]

Additional file 1: Primer sequences for candidate gene microsatellites

Gene	Designator	Forward/Left Primer	Reverse/Right Primer	Product Size
***ARX**	First Set	GAATCGCTCCAAAGTTGCAC	ACCAAACCTCCTCCCAATTT	245
***CACNA1A**	First Set	AAAACCACCATTCTGCATCC	TTCCTTTCCTTCCTCCACCT	168
***CACNA1A**	Second Set	GGAAACACACCAACCCTCAT	TCGAGGAAGGGCTGTCTAAA	156
***CACNA1A**	Third Set	TGGGTGTTTGTCCTCTTTCC	TGTTCGCTTCATGATTGGTC	214
***CACNA1A**	Fourth Set	CCTGCTTCTCCCTCTGTCTG	GCACCTGGATACAGCCATTC	190
CACNA1B	First Set	GCTTCTCCCTCCTCCTGTCT	TCCTTTGCTCTTGGCTCTTC	165
CACNA1D	First Set	AATACACCCAGGGCAATTCA	TAAGCCAGATCCCAGCTGAT	364
CACNA1E	First Set	TTAATGAGGCGTGCATGTGT	CAAACCCTCCAGGACTTTCA	303
CACNA1F	First Set	AACTTCCTTCTTCGGGGATG	TCATGATCTTGGGGTTGTGA	400
CACNA1G	First Set	TTCCATCCATCCGTCTATCC	CCACGTTGCTAGCTTGATGA	237
CACNA1G	Second Set	CAGACACAACTGCTCACACG	GCCTGTTGGAATCTTTGGAA	166
***CACNA1H**	First Set	CTGCTGTGGCTGAAGTGGTA	AGTTCTCGGAGACCAAGCAG	346
***CACNA1H**	Second Set	AGTACGCATTGGAAGAAGCAG	GGTCAAGCAACTTGTTCTAGGTC	250
CACNA1I	First Set	CTTCTCCCTCTGCCTGTGTC	TCCCCTTTTGACTTGATTGC	239
**#CACNA2D2**	First Set	TTGGCAGAGGAATCTTCACC	GTATTCAGCAGGGAGGAGCA	214
**#CACNA2D2**	Second Set	GAGTCCAATGTCAGGCTTCC	AGGGAATAACTGGCATGCTG	164
CACNB1	First Set	GCGCTCAGTCCTCAATAACC	AGCTCGAAGATTCGCTCAAT	302
CACNB2	First Set	TCTCAACTGCCAGAGAGCAG	TGCCCATGTCTCTGACTATCC	454
CACNB3	First Set	GGATGGCGTGTATGATGTTG	AGATGCCTGGTTTGCTGACT	411
***CACNB4**	First Set	GCCCTTCTCCTCTGCTCATT	GCTCGAGATTCGTGAAAAGG	246
**#CACNG2**	First Set	GATGGGGACCTTTCATCTTG	TGGTGCTGGAGAAAATCTGA	244
CACNG3	First Set	CAATCTGGGAGCGTGTCTCT	TCTCCTGCTCAACCTTCCTC	322
CACNG3	Second Set	GTGTGGGAAGGTAAGCCAGA	ATCACCTCCAAACACCAAGC	330
CACNG4	First Set	CGGGATTCTGAAATCTCCTG	TTCTCCTCAGGATCGCTGTT	357
CACNG6	First Set	CCTGTGTTGGGCTCCATAAG	CTGGGCACCCCTTTTATTTA	181
CHRNA1	First Set	CCCCAAGAAGTGCAAGAAGA	TGTCATGGGATTATGGGTCA	158
CHRNA1	Second Set	TGCTTCTGCCTGTGTCTCTG	GGCTCCTGGTTTAGTTGGTG	199
CHRNA1	Third Set	CCCAGCAACAAAACAAAATG	CTTCTCCCTCTGCCTGTGTC	183
CHRNA1	Fourth Set	AGGGGCAAACATAGGAACAG	CTTCTCCCTCCGTCTGTGTG	241
***CHRNA2**	First Set	CCTCCAGAGAGGGTGTGTGT	TCATGCAGGTGCTACAGAGG	244
CHRNA3	First Set	TTCAACCCCTTTACCCATCA	TTTCCAAAGCAATCACACCA	327
***CHRNA4 (and KCNQ2)**				
CHRNA5	First Set	TGATCTTGGGGTCATGAGTTC	AAGGGTCCCAATTTCTCCAC	196
CHRNA7	First Set	TATCCTGGACACCCTGGAAG	GCATTACAAGAGGGGAAAGG	191
CHRNA9	First Set	TGAGAGGAGCTCATGGACAA	TCCATGCAAGACAGCATTTC	172
CHRNA10	First Set	TTTCTCTCCCTTTCCCCTTC	TCAGCAGAGGGCATAGCATA	168
CHRNB1	First Set	GAGACAAAATGGGGAGGACA	CTCTTCTCCACCTCCCTCCT	164
***CHRNB2**	First Set	GCTTTATTGCCATGGTCTGG	GATGCCCTGCTCATCCTATG	325
***CHRNB2**	Second Set	AGCTGGTGAGTGGAAGAACC	GCAGATCATGACCACCAATG	215

***CHRNB2**	Third Set	GAAGACCCGGATAGAGTCC	GAGCCAATTCTTTGTTTCATTG	195
CHRND	First Set	TGGTTCCATGCCTTTCTTTC	GACATCTGGGGAGAGAGTGC	316
***CLCN2**	First Set	TCCATCGAGTTTAATCCTTCTTTC	CTCTCTCCTTCTCCCACTGC	160
DNM1	First Set	TAGGCTTTGCCAAGTGAGTG	TCCCTGAAGCCTTCTTCTTG	235
DNM1	Second Set	TCATGTGTGCGCTCACTCTC	GCATCAGTTCTGGTTTGTTGG	196
DNM1	Third Set	TGCCAACTGGGAGACAGACA	TTTGAGAACCCACGAAAGCA	329
DNM1	Fourth Set	CCAAAACCCCATCTTTCTATCC	TTGCTTGCTCTTCAGCAGTG	127
DNM1	Fifth Set	GTGGTGATGCAATGCCTGAT	AGCTGTGTGACCTGGGGACT	382
***GABRA1**	First Set	AGCATGACAGTCCCATCTCC	GGCTGTGAATGATGATGAGC	250
***GABRA1**	Second Set	CAATTCTTGTTTTGGCTAGTGG	AACATTTTCCTCCATAGATACCTTTC	241
***GABRA1**	Third Set	AAACAAGAAAAGCCTGTTGG	AGTCACTTTGGGGAATGTGG	372
GABRA2	First Set	TGACAGTTAAAATTGCGAGGA	TGGCATTTTTCTTTCCTGCT	159
GABRA6	First Set	CAGCAGTGGCTGAATGACA	ACTGCCTACCTCCACCCTTT	339
***GABRD**	First Set	CCTGCTTCTCCCTCTGTGTC	ATTCCCTGGCATGCTATCTG	157
***GABRD**	Second Set	AGAACCACAAAAGCCAGTCC	CCCTCTGCCTATGTCTCTGC	233
***GABRD**	Third Set	CACCCTCCTAGCTGTTCTGC	TGTCCACAGCTTCATTGTCC	205
***GABRG2**	First Set	TCTTCTCCAACCCTCCCTCT	TGCTTTGGTTTCCTTACTGG	152
***GABRG2**	Second Set	TCCTCTGACACATCTAGCAAGC	AAAGGGCAGATGTGTGTGTG	182
***KCNA1**	First Set	TTCCTTTCCGTTTGAATTGC	CAAAAGTGGGAATGGGTCTG	399
KCND2	First Set	TTGGTGGCACGTAAGTTCAG	GTGAGTTCAAGCCCCACAAT	224
KCND2	Second Set	GGCAGGCTTGAATGTTTGAG	AGGGCCTTTGTTCCTAGACG	247
KCND2	Third Set	GAGCCTGCTTCTCCCTCAG	TCAGGAGGGCCAGTTTATTAC	244
KCNQ1	First Set	GTGCAGCATGATGAGTTTGG	GAGGGGTCTAAAGCCCTGAC	367
***KCNQ2 (and CHRNA4)**	First Set	ACCCCCAGACCAACTCTCTT	AGAGCTGCCAATGACAAGGT	163
***KCNQ2 (and CHRNA4)**	Second Set	TAAGGGACTTGGAGCCAGAC	ATGTGTTGGGGACCGAAAG	161
***KCNQ2 (and CHRNA4)**	Third Set	CCCACTCCACAACCTCTCTG	GAGAGCTGCCAATGACAAGG	214
***KCNQ2 (and CHRNA4)**	Fourth Set	GAGCCTGACCGATACGAGAG	AGGTTCAGCAGGGAGAACTG	391
***KCNQ3**	First Set	CTCCTCAGCTGACCTTCGAG	GATTGTACCTGGCCCAACTG	334
KCNQ5	First Set	GGGGAGGCAGGTATAAAGGA	TTGTTGAAAACTACAGGTTATTCAGG	164
***LGI1**	First Set	GCACCCTGAAATGGCATAGT	TGCCCTCTAGCTTCCTTCAA	151
***LGI1**	Second Set	CCTGGCCATCAAATAAATCC	AGCCAAAGGCAGACACACTC	325
***ME2**	First Set	CTCTCCCCTTTAGGCAGCTT	CCCTCTGCCTATGTCTCTGC	196
***NHLRC1**	First Set	AAGGTGGCGTTAAACTGCTG	CCGAAAGGGAGGAAAGATTC	366
***NHLRC1**	Second Set	CAATGCTTGCTGCACAGAGT	GTTAACCACCTGCCTTCAGC	327
***SCN1A**	First Set	AACACTGACATTTTCTCGACCT	GCTGTTGAGCATCTGCCTTC	249
***SCN1A**	Second Set	TTTGGAAAGTACGTGGGGTA	CCCTCCTGACTGGACAAAGA	187
***SCN1A**	Third Set	CTGCCTGAACTGGGTATGTG	CCTGGGAACACTACCTGAGC	201
***SCN1A**	Fourth Set	TCAAACAAAATTGGGGTTCC	ACCTCAGGCAGATTGATTGG	309
***SCN1B**	First Set	TGGAAAGGAGAGGTGAGGTG	AATGCCTACCCTGCAGTCAG	401
***SCN1B**	Second Set	GCCCCAGCACTGTGTTTATC	AAACTCTTGATGGCCCTGTG	246
***SCN1B**	Third Set	CTTCTCCCTCTGCCTGTGTC	CAGGCGAAGAGCTCAGTAGG	188

***SCN1B**	Fourth Set	CCCTAAGTAGGGGCCAAGAG	TTTGAGGAGGGATGTGGAAC	347
***SCN2A**	First Set	AAGCTTGCAGTGGCTCATTC	GGGTGGCTCAGTTGGTTAAA	468
SCN3A	First Set	AATCACTTCCATGCATTCCA	CTGCCTGTTTCTCTGCCTCT	359
SCN3A	Second Set	TTATGGAAAGAGCCCACCTG	CTCCCCTCTGGTAACCATCA	317
SCN3B	First Set	CACCAGGTGCAGGACTACAA	TAGAGCCAGGAGGTCAGAGC	394
SCN3B	Second Set	CTGATGCCGTTCTGAAGGTT	TCGAATGCCCTGAGAGAAGT	339
SCN8A	First Set	CTGGAGACGTGGGATCAAGT	TCCTTCCTTCCTTCCCTTTC	311
SCN8A	Second Set	AAAGTCAGGCCAACAAATAGG	TCCTCCTCCTCCTTCTCCTC	250
SCN11A	First Set	CACGCTGAGCATGGAAACTA	AAGCAAATGGCTGAACCAAG	432
