# Supplementary material for: Candidate genes for idiopathic epilepsy in four dog breeds
Source: BMC Genet. 2011 Apr 25;12:38. doi: 10.1186/1471-2156-12-38 (PMC3111397; doi:10.1186/1471-2156-12-38)
Supplement: Additional file 2 — Candidate gene locations and primer locations. All pertinent canine chromosomal location information is presented for each gene and all microsatellites examined for that gene. Bolded genes marked with a * indicate those associated with human epilepsy, and bolded genes marked with a # indicate those associated with mouse models of epilepsy. Two of the DNM1 marker primer pairs, DNM1 Third Set and DNM1 Fifth Set, are taken from the University of California-Davis Canine Genetic Linkage Map [74], and are named therein 0945 and 0946, respectively. ^ Bolded entries in this column indicate microsatellites located outside the gene itself and further than 1 Mb away (n = 3). Mb positions given for microsatellites are the first nucleotide of each microsatellite. Mb positions were taken from Ensembl [75] and the UCSC Genome Browser [61]. CFA = canis familiaris chromosome. [file 1471-2156-12-38-S2.PDF]

Additional file 2: Candidate gene locations and primer locations

Gene	Chromosome	Gene Mb Position	Mb Position of MS^	Designator
***ARX**	CFA X	20,498,171-20,508,795	20,490,133	First Set
***CACNA1A**	CFA 20	51,822,487-52,036,979	51,838,435	First Set
***CACNA1A**	CFA 20	51,822,487-52,036,979	51,994,400	Second Set
***CACNA1A**	CFA 20	51,822,487-52,036,979	51,838,981	Third Set
***CACNA1A**	CFA 20	51,822,487-52,036,979	51,792,498	Fourth Set
CACNA1B	CFA 09	51,142,446-51,335,492	51,309,940	First Set
CACNA1D	CFA 20	39,187,585-39,340,565	39,320,710	First Set
CACNA1E	CFA 07	18,012,734-18,224,369	18,042,152	First Set
CACNA1F	CFA X	42,256,561-42,281,312	42,258,442	First Set
CACNA1G	CFA 09	29,805,671-29,868,112	29,826,364	First Set
CACNA1G	CFA 09	29,805,671-29,868,112	29,809,345	Second Set
***CACNA1H**	CFA 06	42,552,039-42,574,199	42,569,081	First Set
***CACNA1H**	CFA 06	42,552,039-42,574,199	42,540,979	Second Set
CACNA1I	CFA 10	28,448,535-28,549,917	28,460,916	First Set
**#CACNA2D2**	CFA 20	41,963,231-41,993,458	41,994,393	First Set
**#CACNA2D2**	CFA 20	41,963,231-41,993,458	41,899,484	Second Set
CACNB1	CFA 09	26,511,656-26,523,270	26,516,837	First Set
CACNB2	CFA 02	21,372,899-21,552,240	21,534,961	First Set
CACNB3	CFA 27	8,716,494-8,724,279	8,694,473	First Set
***CACNB4**	CFA 19	56,021,060-56,208,002	56,068,479	First Set
**#CACNG2**	CFA 10	30,823,171-30,934,371	30,834,662	First Set
CACNG3	CFA 06	24,646,198-24,729,961	24,675,408	First Set
CACNG3	CFA 06	24,646,198-24,729,961	24,695,069	Second Set
CACNG4	CFA 09	16,581,359-16,591,826	16,580,869	First Set
CACNG6	CFA 01	106,304,980-106,320,023	106,305,803	First Set
CHRNA1	CFA 36	21,751,465-21,767,727	21,761,429	First Set
CHRNA1	CFA 36	21,751,465-21,767,727	21,726,792	Second Set
CHRNA1	CFA 36	21,751,465-21,767,727	21,702,419	Third Set
CHRNA1	CFA 36	21,751,465-21,767,727	21,809,937	Fourth Set
***CHRNA2**	CFA 25	33,113,350-33,123,779	33,105,508	First Set
CHRNA3	CFA 13	41,356,174-41,373,509	41,385,716	First Set
***CHRNA4 (and KCNQ2)**	CFA 24	50,071,617-50,092,266	See KCNQ2	
CHRNA5	CFA 13	41,372,365-41,382,783	41,385,716	First Set
CHRNA7	CFA 03	39,708,367-39,725,043	39,831,720	First Set
CHRNA9	CFA 03	75,016,530-75,029,238	75,026,269	First Set
CHRNA10	CFA 21	29,221,050-29,225,988	29,240,190	First Set
CHRNB1	CFA 05	35,371,137-35-378-838	35,335,585	First Set
***CHRNB2**	CFA 07	45,764,643-45,770-670	45,752,217	First Set
***CHRNB2**	CFA 07	45,764,643-45,770-670	45,770,147	Second Set
***CHRNB2**	CFA 07	45,764,643-45,770-670	45,803,287	Third Set
CHRND	CFA 25	47,166,290-47,173,157	47,154,539	First Set
***CLCN2**	CFA 34	20,231,733-20,245,805	20,350,332	First Set
DNM1	CFA 09	58,599,318-58,642,207	58,611,451	First Set
DNM1	CFA 09	58,599,318-58,642,207	58,548,110	Second Set
DNM1	CFA 09	58,599,318-58,642,207	59,305,239	Third Set
DNM1	CFA 09	58,599,318-58,642,207	**59,676,496**	Fourth Set
DNM1	CFA 09	58,599,318-58,642,207	**60,008,822**	Fifth Set
***GABRA1**	CFA 04	52,213,113-52,265,985	52,241,889	First Set
***GABRA1**	CFA 04	52,213,113-52,265,985	52,180,151	Second Set
***GABRA1**	CFA 04	52,213,113-52,265,985	52,306,846	Third Set
GABRA2	CFA 13	45,304,924-45,354,400	45,358,899	First Set
GABRA6	CFA 04	52,413,266-52,429,586	52,435,009	First Set
***GABRD**	CFA 05	59,985,863-59,996,203	59,979,940	First Set

***GABRD**	CFA 05	59,985,863-59,996,203	59,970,925	Second Set
***GABRD**	CFA 05	59,985,863-59,996,203	60,007,128	Third Set
***GABRG2**	CFA 04	52,000,524-52,105,089	52,047,673	First Set
***GABRG2**	CFA 04	52,000,524-52,105,089	52,051,927	Second Set
***KCNA1**	CFA 27	42,963,751-42,965,238	42,952,301	First Set
KCND2	CFA14	61,864,675-61,880,458	61,527,738	First Set
KCND2	CFA 14	61,864,675-61,880,458	61,729,874	Second Set
KCND2	CFA 14	61,864,675-61,880,458	61,817,857	Third Set
KCNQ1	CFA 18	49,613,783-49,782,796	49,696,953	First Set
***KCNQ2 (and CHRNA4)**	CFA 24	50,107,441-50,135,495	50,187,606	First Set
***KCNQ2 (and CHRNA4)**	CFA 24	50,107,441-50,135,495	50,257,839	Second Set
***KCNQ2 (and CHRNA4)**	CFA 24	50,107,441-50,135,495	50,187,606	Third Set
***KCNQ2 (and CHRNA4)**	CFA 24	50,107,441-50,135,495	49,884,851	Fourth Set
***KCNQ3**	CFA 13	31,810,606-31,858,601	32,041,764	First Set
KCNQ5	CFA 12	38,194,576-38,360,861	38,004,201	First Set
***LGI1**	CFA 28	10,964,801-11,003,562	10,985,774	First Set
***LGI1**	CFA 28	10,964,801-11,003,562	10,944,519	Second Set
***ME2**	CFA 01	27,016,792-27,062,785	27,022,785	First Set
***NHLRC1**	CFA 35	19,939,702-19,940,841	19,945,196	First Set
***NHLRC1**	CFA 35	19,939,702-19,940,841	19,933,933	Second Set
***SCN1A**	CFA 36	14,120,290-14,195,687	14,163,331	First Set
***SCN1A**	CFA 36	14,120,290-14,195,687	14,182,019	Second Set
***SCN1A**	CFA 36	14,120,290-14,195,687	14,223,131	Third Set
***SCN1A**	CFA 36	14,120,290-14,195,687	14,263,124	Fourth Set
***SCN1B**	CFA 01	120,412,274-120,419,152	**118,811,176**	First Set
***SCN1B**	CFA 01	120,412,274-120,419,152	120,385,266	Second Set
***SCN1B**	CFA 01	120,412,274-120,419,152	120,428,964	Third Set
***SCN1B**	CFA 01	120,412,274-120,419,152	120,462,087	Fourth Set
***SCN2A**	CFA 36	13,532,170-13,615,197	13,544,296	First Set
SCN3A	CFA 36	13,312,004-13,387,729	13,323,890	First Set
SCN3A	CFA 36	13,312,004-13,387,729	13,380,999	Second Set
SCN3B	CFA 05	13,789,622-13,809,096	13,802,474	First Set
SCN3B	CFA 05	13,789,622-13,809,096	13,819,946	Second Set
SCN8A	CFA 27	6,179,810-6,298,254	6,278,708	First Set
SCN8A	CFA 27	6,179,810-6,298,254	6,254,074	Second Set
SCN11A	CFA 23	11,569,985-11,654,091	11,590,625	First Set
